# A Great Mistake

**Published:** 1866-12

**Authors:** 


### A GREAT MISTAKE.

Many persons precipitate themselves into the grave by attempting to bravado an ailment; to be up and about in defiance of it. If anything at all is the matter with a man which is really disquieting, he should at least have as much sense as a pig, and go and lie down; pigs are not such fools as to move about in pain; it is a great deal better to lie down and

